# Role of Percutaneous Ablation in the Management of Intrahepatic Cholangiocarcinoma

**DOI:** 10.3390/medicina59071186

**Published:** 2023-06-22

**Authors:** Georgios Charalampopoulos, Roberto Iezzi, Maria Tsitskari, Argyro Mazioti, Olympia Papakonstantinou, Alexis Kelekis, Nikolaos Kelekis, Dimitrios Filippiadis

**Affiliations:** 1 2nd Department of Radiology, University General Hospital “ATTIKON”, Medical School, National and Kapodistrian University of Athens, 12462 Athens, Greece; charalampopoulosg@outlook.com (G.C.); argyromazioti@yahoo.gr (A.M.); sogofianol@gmail.com (O.P.); akelekis@med.uoa.gr (A.K.); kelnik@med.uoa.gr (N.K.); 2 Department of Diagnostic Imaging, Oncologic Radiotherapy and Hematology, A. Gemelli University Hospital Foundation IRCCS, Catholic University of the Sacred Heart, 00168 Rome, Italy; roberto.iezzi.md@gmail.com; 3 Apollonio Private Hospital, 20 Lefkotheou Avenue, 2054 Strovolos, Nicosia, Cyprus; mariadote@hotmail.com

**Keywords:** intrahepatic cholangiocarcinoma, ablation, cancer

## Abstract

Cholangiocarcinoma (CCA) is an invasive cancer accounting for <1% of all cancers and 10–15% of primary liver cancers. Intrahepatic CCA (iCCA) is associated with poor survival rates and high post-surgical recurrence rates whilst most diagnosed patients are not surgical candidates. There is a growing literature suggesting percutaneous ablative techniques for the management of patients with iCCA measuring ≤3 cm with contraindications to surgery as well as for recurrent or residual tumors aiming to provide local cancer treatment and control. Most used ablative therapies for iCCA include radiofrequency and microwave ablation with irreversible electroporation, cryoablation and reversible electroporation (electrochemotherapy) being less commonly encountered techniques. Due to the infiltrative margins of the lesion, there is a need for larger safety margins and ablation zone; multi-apparatus ablation or other variations of the technique such as balloon-assisted approaches can be utilized aiming to increase size of the zone of necrosis. The present review paper focuses upon the current role of percutaneous ablative techniques for the therapeutic management of iCCA. The purpose of this review is to present the current minimally invasive ablative techniques in the treatment of iCCA, including local control and survival rates.

## 1. Introduction

Cholangiocarcinoma (CCA) is an aggressive primary malignancy arising from the epithelial cells of the biliary tract and is the second most common primary liver cancer after hepatocellular carcinoma (HCC) [1]. CCA is an invasive cancer and accounts for <1% of all cancers and 10–15% of primary liver cancers and is subclassified anatomically as intrahepatic (iCCA), arising proximally to the second-order bile ducts, and extrahepatic (ECC), arising from the extrahepatic bile ducts [2,3]. The growth pattern of iCCA can be mass forming, periductal-infiltrating and intraductal [4,5]. The overall incidence and mortality rates of iCCA has been increasing in the last decades globally [6,7] and the highest incidence and mortality rates were among males, patients older than 65 years and Asians [8].

CCA is most caused by chronic inflammation and cholestasis [9]. Risk factors associated with iCCA development are cirrhosis, hepatitis B virus (HBV) and hepatitis C virus (HCV), primary sclerosing cholangitis (PSC), hepatolithiasis, hepatobiliary flukes, biliary cirrhosis, gallstones, choledochal cysts, Caroli’s disease, chronic infection by Salmonella typhi or Helicobacter bilis, metabolic syndrome, non-alcoholic steatohepatitis and obesity [10,11,12,13,14,15]; however, the majority of iCCA cases are not associated with any risk factors [16]. iCCA is associated with a poor prognosis and a high mortality rate due to its insidious and aggressive nature, with many patients presenting with locally advanced disease and even metastases, thus limiting therapeutic options [17,18]. Surgical margin-negative resection is considered the only definite treatment, but only about 20% of cases are candidates for surgical treatment [3,19] because of an advanced stage at the time of diagnosis, comorbidities or advanced age [20,21]. After surgical treatment, median survival approximates 28–30 months [22], 3-year overall survival is 52–68.6% in complete resection with negative margins [23,24] and 5-year survival rate is 23–42%, while it does not exceed 5% in inoperable cases [25]. The majority of iCCA cases require extended liver resection [26] but recurrence rates after resection are high, reaching 60–65% [6] at a median of 18 months [27] with risk factors for recurrence being metastases to lymph nodes, vascular invasion, multifocal iCCA, low histologic grading and size of the tumor [28]. Studies have identified several herbs and formulas that exhibit potential anti-tumor effects against iCCA, including Curcuma wenyujin, Salvia miltiorrhiza, Bufei Yishen formula and Jianpi Huaji formula; these herbs and formulas have been shown to inhibit cancer cell proliferation, induce apoptosis, and regulate immune function [29,30].

Due to the complex treatment management of iCCA, interdisciplinary discussion and close co-operation among hepatobiliary and transplant surgeons, interventional oncologists, endoscopists and medical oncologists is mandatory for a patient-tailored approach [31]. In most iCCA cases, deaths are related to local disease progression rather than metastases [32]; thus, locally ablative therapies are becoming increasingly important in the management of iCCA [3,33,34]. A growing literature supports the application of percutaneous ablative therapies to iCCA; in a recent systematic review and pooled analysis upon locoregional therapies for iCCA, the results of only ablation were sufficiently consistent to allow for recommendation of use in non-surgical candidates [35,36]. Compared to surgical approaches, the rationale for proposing percutaneous ablation includes the fact that the technique is governed by a minimally invasive approach which is well tolerated even in patients with co-morbidities or with extensive disease related to a low overall morbidity rate. Furthermore, complication rate, cost, hospitalization time, blood loss and procedure time favor percutaneous over surgical approaches.

The present review paper focuses upon the current role of percutaneous ablative techniques for the therapeutic management of iCCA. This is not a systematic review of the literature. Several separate literature searches were performed. Non-English studies and case reports were excluded from the study. All references of the obtained articles were also evaluated for any additional information. The purpose of this review is to present the current minimally invasive ablative techniques in the treatment of iCCA, including local control and survival rates.

## 2. Interventional Ablative Treatment for iCCA

Ablative therapies for iCCA consist of mainly of radiofrequency (RFA) and microwave ablation (MWA), (Table 1) whilst cryoablation, irreversible electroporation (IRE) and electrochemotherapy constitute newer approaches.

Ablation should be considered for patients with iCCA measuring ≤3 cm who have contraindications to surgery [3] and also in recurrent or residual tumors [21] to provide local cancer treatment and control. In comparison to surgical treatment, ablation is cheaper and simpler to perform with a low rate of complications and reduced hospital stay [37]. Compared to palliative treatments with systemic therapies, percutaneous ablation has been shown to lead to favorable clinical outcome in case of recurrent iCCA [27].

**Table 1:** Thermal ablation in the treatment of ICCA.

Study	Treatment Modality	Patients	Lesions	Follow-Up (Months)	OS 1 yr	OS 2 yr	OS 3 yr	OS 4 yr	OS 5 yr	Technical Efficacy	Complication	Minor Complication Rate	Reference Number
Chiou et al., 2005	RFA	10	10	20 (4–38)	100%					80%	0%	50%	[38]
Carrafiello et al., 2010	RFA	6	6	17.5 (13–21)						66%	0%	16.7%	[39]
Kamphues et al., 2010	RFA	13	17	28 (12–69)	92%		52%		83.3%	66.7%	0%	7.6%	[40]
Giorgio et al., 2011	RFA	10	10	19.5 (9–64)	100%		83.3%				0%	30%	[41]
Kim et al., 2011	RFA	13	17	19.5 (3.3–82.1)	85%		51%		15%	88.2%	5.9%	41.2%	[42]
Yu et al., 2011	MWA	15	24	12.8 (4–31)	60%					87.5%	20%	60%	[43]
Fu et al., 2012	RFA	17	26	29 (4–122)	84.6		43.3		28.9	96.2%	3.6%	28.6%	[44]
Haidu et al., 2012	RFA	11	36	35 (12–81)	91%		71%			91.7%	13%		[45]
Xu et al., 2012	RFA, MWA	18	25	8.7 (1.3–86.2)	36.3		30.3		30.3	92.%	5.6%	5.6%	[46]
Zhang et al., 2013	RFA, MWA	77	133	26.7 (5.0–66.7)	69.8%		20.5%			94.7%	3.9%		[47]
Yang et al., 2015	MWA	26	39	19.2 (6–30)	69.2%	61.5%				92.3%	0%	88.5%	[48]
Zhang et al., 2018	MWA	107	171	20.1 (2.8–63.5)	93.5%		39.6%		7.9%	92.9%	2.8%		[49]
Giorgio et al., 2019	RFA, MWA	71	98	48 (8–86)	88%		65%		45%		0%		[50]
Ni et al., 2019	MWA	78	106	22.7 (1–86.7)	89.5%		52.2%		35%		3.8%	29.5%	[51]
Wu et al., 2019	RFA	86		12.3 (6.5–25.1)					17.6%				[52]
Xu et al., 2019	MWA	56	62		81.2%		42.5%		23.7%	100%	5.4%		[53]
Brandi et al., 2020	RFA	29	117	39.9 (2–55)	89%	45%		11%		92.3%	6.8%	14.4%	[32]
Wang et al., 2020	RFA, MWA	77	226	14 (4–69)	69.6%		29.5%		23.6%		1.3%	32.5%	[54]
Diaz-Gonzalez et al., 2020	RFA, MWA	27	33	26.5 (20.2–45.7)	88.9%		40.7%		14.8%	92.6%	3.7%	11.1%	[55]
Xiang et al., 2020	RFA	34	34	31 (25–34)	89.9%		42.4%		23.9%				[56]
Braunwarth et al., 2020	RFA	11	11	16 (1–116)	93.8%		71.6%		47.7	90,9%			[27]
Yang et al., 2021	MWA	52	74	21.2 (3.2–78.7)	87.4%		51.4%		56.9%	100%	3.8%	3.8%	[57]
Chu et al., 2021	RFA	40	64	26 (3.3–132)	67.2%		36.2%		18.3	96.9%	7.5%		[58]

From a technical point of view and due to the infiltrative margins of iCCA lesion a minimum of 1 cm is necessary for safety margins. As a result, larger ablation zones are necessary for an effective ablation of iCCA. To achieve these larger ablation zones, either multi-apparatus or balloon-assisted ablation have been utilized [59]. Triple-antenna MWA have been shown to result in the creation of a reliable 6 cm ablation zone in the liver [60], whilst balloon assisted ablation combines use of a micro balloon catheter and balloon inflation with resultant flow redistribution followed by thermal ablation [59].

### 2.1. Radiofrequency Ablation

RFA is a thermal ablative method which uses high-frequency alternating electric current to generate frictional heat through rapid electron vibration resulting in coagulative necrosis of tissue [61]. It is heavily dependent on good tissue thermal conductivity which in turn is correlated to the water content of the tissue [61,62]. At the temperature of 100 °C, the tissue adjacent to the tip of the probe heats up and becomes desiccated. The desiccated tissue then acts as an “insulating sleeve” surrounding the probe and limits further transmission of thermal and electrical energy and limits the size of the ablation zone [61]. Heat is also lost by the cooling effect of flowing blood within adjacent blood vessels which act as a “heat sink” limiting the ablation zone and potentially leaving behind residual unablated cancerous tissue [61,63]. To achieve a complete tumor coverage for larger lesions, including a safety margin of 0.5 to 1 cm, multiple RFA probes can be used to augment the ablation zone size [27,45,64].

RFA is considered effective if a complete tumor ablation is achieved and efficacy is closely associated with tumor size with achievement of complete necrosis in nearly all tumors smaller than 3 cm with significantly lower results for larger lesions [39,41,42,56]. Success rate of RFA is also affected by tumor location and surrounding tissue, with centrally located tumors being difficult to be treated due to “heat sink” effect of the adjacent vessels in the liver hilum [65]. A 2021 meta-analysis revealed a technical efficacy of thermal ablation techniques (RFA and MWA) between 66.7 and 100.0% with a pooled technical efficacy of 91.9% (95% CI, 87.3–94.9%) [66].

A 2015 meta-analysis of RFA in the treatment of iCCA revealed pooled 1-year, 3-year and 5-year survival rates of 82% (95% CI, 72–90%), 47% (95% CI, 28–65%) and 24% (95% CI, 11–40%) [67]. A retrospective study including 29 patients revealed median overall survival (OS) of 89%, 45% and 11% at 1, 2 and 4 years, respectively, with a median local tumor-progression-free survival of 9.27 months for all tumors but significantly longer in patients with tumor size less than 20 mm [32]. RFA is associated with significantly better survival rates in patients with unresectable tumors compared with varied palliative treatment as was shown in previous studies [38,42,52,68]. Several studies that assessed the efficacy of RFA treatment for patients with recurrent ICC show favorable results with a median overall survival similar to repeated resection, indicating that ablation can be an effective treatment for these patients [44,69] but with tumors less than 3 cm [47] and repeated resection can be safely combined with RFA with good results [40]. For large tumors sequential multiple sessions can be used [54], and stereotactic RFA with the creation of overlapping ablation zones can also be an option to extend survival [27,70]. For small early-stage primary ICC, surgical resection provides a significantly better prognosis than RFA and is still recommended as the first-line treatment, but there is a need for prospective randomized controlled trials to assess this [56]. 

RFA is considered a safe technique with less complications compared to surgical resection [37,67], including liver abscess, biloma, biliary stricture, bleeding, pleural effusion, post-ablative syndrome [32,44,54,67,69] and a risk of tract seeding which is decreased with tract ablation [43].

### 2.2. Microwave Ablation

Microwave ablation is an alternative to RFA thermal ablative techniques where electromagnetic waves are used to induce rotation of dipole water molecules, resulting in frictional heat and, thus, inducing cellular death via coagulation necrosis [71]. MWA has potential advantages over RFA such as shorter ablation and operative time, higher temperatures in a short time achieving larger ablation zones, less susceptibility to cooling heat-sink effects of adjacent blood vessels and effective in the high-impedance desiccated tissue in which RFA would not be successful [72,73].

**Figure 1:**
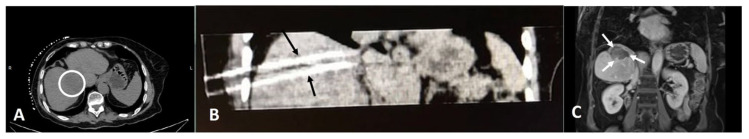
(**A**). A 64 y-o female patient with a post-surgical recurrent solitary mass of intrahepatic cholangiocarcinoma. CT axial scan with no IV injection of contrast medium at the beginning of ablation session illustrating the hypodense iCCA mass (white circle). (**B**). Coronal CT reconstruction illustrating two microwave antennas (black arrows) placed in the mass. (**C**). MRI 12 months post ablation (TI-weighted sequence with fat signal suppression post IV injection of Gadolinium illustrates the zone of necrosis (white arrows).

A few studies assessing MWA treatment of iCCA (Figure 1) have been published and the largest retrospective study assessing patients with primary or recurrent ICCA demonstrated median OS of 28 months, OS at 1, 3 and 5 years of 93.5%, 39.6% and 7.9%, respectively, and median progression-free survival of 8-9 months with no procedure-related deaths and a 2.8% frequency of major complications [49]. Overall survival was associated with the number of tumors [49]. Favorable outcomes were also found in another study which reported ablation success rate of 91.7%, technique effectiveness rate of 87.5% and local tumor progression rate of 25% with OS rates at 6, 12 and 24 months of 78.8%, 60.0% and 60.0%, respectively [43]. Safety and efficacy of MWA in the treatment of iCCA was also shown in a single-center retrospective study which demonstrated cumulative OS at 1, 3 and 5 years of 87.4%, 51.4% and 35.2%, respectively, and cumulative recurrence free survival at 1, 3 and 5 years of 68.9%, 56.9% and 56.9%, respectively, while the major complication rate was 3.8% [57]. A 2019 study comparing MWA to surgical resection for recurrent iCCA concluded that there was no significant difference in 5-year OS between the groups (23.7% in MWA group and 21.8% in surgical resection group) while complication rate, cost, hospitalization time, blood loss and procedure time were better in the MWA group [53]. MWA was considered a comparable and valid alternative to surgical resection in the treatment of recurrent iCCA [53]. MWA was compared to RFA in treatment of unresectable iCCA in a 2019 retrospective study which found that MWA was superior to RFA in achieving better long-term survival with tumors up to 3 cm as well as tumors up to 4 cm and better local tumor control [50]. MWA can be also combined with other locoregional therapies such as trans-arterial conventional chemoembolization, as was shown in a retrospective study which reported survival at 6, 12 and 24 months of 88.5%, 69.2% and 61.5%, respectively [48]. Furthermore, stereotactic navigation systems contribute to shortening the duration of ablation sessions by increasing the accuracy of access, even in challenging anatomic locations of the liver or for tumors located in proximity to important vasculature as well as for tumors invisible on ultrasound and CT [74,75]. 

Thermal ablative therapies (RFA and MWA) display promising potential as treatment modalities for iCCA with satisfactory outcome and good safety profile as was shown in published meta-analyses and systematic reviews [35,66,73] showing pooled technical efficacy of 91.9%, major complications incidence of 5.7% and pooled OS at 1, 3 and 5 years of 82.4%, 42.1% and 28.5%, respectively, with primary tumors showing higher 3-year OS rates than recurrent ones [66]. Tumor size > 3 cm, multiple tumors and age > 65 years were factors associated with shorter OS; thus, thermal ablation is suggested especially for a single iCCA smaller than 3 cm [66].

### 2.3. Irreversible Electroporation

**Figure 2:**
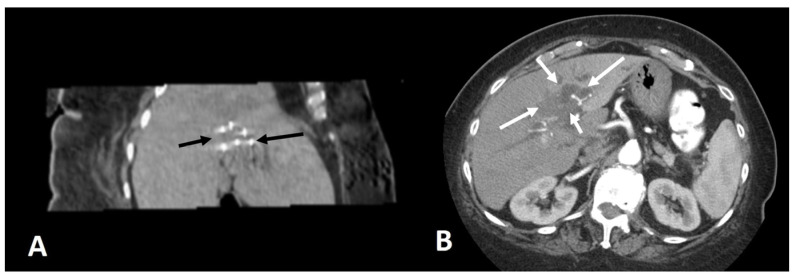
(**A**) A 59 y-o male patient with a post-surgical recurrent solitary mass of intrahepatic cholangiocarcinoma. Coronal CT reconstruction during ablation illustrates 6 IRE needles (black arrows) placed at the mass. (**B**) CT axial scan post IV injection of contrast medium (arterial phase) illustrates the zone of necrosis (white arrows) 3 months post ablation with no signs of remnant or recurrent tumor.

Irreversible electroporation (IRE) is a relatively new non-thermal ablative technique using high-voltage electrical current to create nanoscale holes in the cell membranes to induce apoptotic cell death [76,77]. Specifically for IRE, general anesthesia with complete neuromuscular blockade and electrocardiogram synchronization is considered a prerequisite, as the high voltage that it is used during the procedure causes muscular contraction and potentially cardiac arrhythmias [78]. Advantage of the technique is that it can be safely used for ablation of central tumors located adjacent to sensitive to thermal damage areas [79] and it has been used as a treatment option for centrally located liver tumors with margins adjacent to major bile ducts where thermal ablation techniques are contraindicated [80]. There is a lack of published data regarding iCCA treatment with IRE [81,82,83] and a recent prospective study showed local disease control with a decrease in the entire volume of the lesion and a further reduction of the densitometric values [83], suggesting that IRE should be considered in the treatment of iCCA (Figure 2).

### 2.4. Electrochemotherapy

Electrochemotherapy (ECT)—reversible electroporation—is an emerging non-thermal ablative procedure with approved indication for subcutaneous and cutaneous lesions mainly in head and neck or breast structures. New developments are focused on the treatment of deep-seated tumors [84]. ECT works by increasing the permeability of tumor cells by means of a locally generated electrical field (electroporation), thus rendering the cellular membranes more permeable to hydrophilic chemotherapeutic agents (i.e., bleomycin) [85]. Preliminary single-center experience including five patients only seems to demonstrate its feasibility, safety and effectiveness for improving prognosis and quality of life of patients with unresectable peri hilar CCA [86]. When compared to IRE, being characterized by reduced field strength, ECT could also be performed under analgo-sedation, without significant pain or adverse events as well as complications and without the need of general anesthesia, allowing for rapid post-interventional recovery [87].

### 2.5. Cryoablation

Cryoablation is a technique using an ice ball with temperatures as cold as −160 °C to produce intra- and extracellular ice crystals which cause osmotic pressure changes and dehydration, damaging the cell membrane and leading to cellular death [88]. This technique has the advantages of visibility of the ice ball in CT and MRI imaging and in ultrasonography, it can be applied to treat tumors adjacent to structures sensitive to thermal damage, it is affected to a lesser degree than thermal ablation by “heat sink” effects and cold has analgesic properties. A potential but extremely rare and fatal cryoablation-related complication is cryo-shock, which is largely dependent upon the volume of the destroyed tissue [89,90,91] and thus can be avoided by setting a targeted tumor volume limit in each cryoablation session [92]. Only few studies [93,94] have been published assessing the use of cryoablation for iCCA treatment; thus, current evidence is insufficient to establish the role of cryoablation in these patients.

## 3. Combining Percutaneous Ablative Techniques with Systemic Therapies

Median overall survival of systemic therapy for iCCA when performed as the only therapeutic modality ranges from 11.7 to 15.4 months [95]. Braunwarth et al. have shown that, specifically for recurrences of iCCA, aggressive local curative therapies outperform systemic palliative approaches in terms of 5 years overall survival (47.7% versus 12.3%, respectively) [27]. Case reports illustrate a tendency towards combining local ablative techniques and systemic therapies for the management of advanced iCCA in non-surgical candidates [96,97]. Yan et al. performed a propensity score matching analysis for patients with unresectable ICC who received thermal ablation plus chemotherapy or chemotherapy alone as the initial treatment; the authors concluded that the median overall survival of patients undergoing combine therapy was significantly longer (16.267 versus 6.067, *p* = 0.000), illustrating the benefits of ablation plus chemotherapy to the provision of an opportunity to improve the prognosis of patients with unresectable ICC [98]. Seidensticker et al. suggest to focus on distinguishing supplementation of systemic therapy with either local techniques which lead to complete necrosis (ablation) versus those leading to partial remission [radioembolization or trans-arterial chemoembolization (TACE)] [99]. 

## 4. Conclusions

Intrahepatic cholangiocarcinoma is an aggressive tumor with poor prognosis and high recurrence rates post resection. International guidelines provide recommendations for use of ablation in iCCA patients who are non-candidates for operation, those refusing surgery or with surgical recurrences and a tumor diameter <3 cm. A large zone of necrosis with >1 cm safety margins is necessary. Utilization of microwaves, multi-apparatus or balloon-assisted ablation contribute to the large ablation zone required. Technological developments and clinical application of new techniques could also potentially improve the role of interventional oncology in this field of interest, offering new potential treatment options with curative or palliative aims.

## Data Availability

Not applicable.
